# EGFR Q787Q Polymorphism Is a Germline Variant and a Prognostic Factor for Lung Cancer Treated With TKIs

**DOI:** 10.3389/fonc.2022.816801

**Published:** 2022-03-21

**Authors:** Wen-Jui Wu, Sheng-Hsiung Yang, Hsin-Pei Chung, Chia-Te Yen, Yen-Ting Chen, Wei-Chin Chang, Jian Su, Hsuan-Yu Chen

**Affiliations:** ^1^ Chest Division, Department of Internal Medicine, MacKay Memorial Hospital, Taipei, Taiwan; ^2^ Ph.D. Program in Translational Medicine, National Taiwan University and Academia Sinica, Taipei, Taiwan; ^3^ Department of Pathology, MacKay Memorial Hospital, Taipei, Taiwan; ^4^ Department of Pathology, MacKay Medical College and MacKay Junior College of Medicine, Nursing, and Management, Taipei, Taiwan; ^5^ Institute of Statistical Science, Academia Sinica, Taipei, Taiwan; ^6^ Genome and Systems Biology Degree Program, National Taiwan University, Taipei, Taiwan; ^7^ Ph.D. Program in Microbial Genomics, National Chung Hsing University, Taichung, Taiwan

**Keywords:** EGFR, Q787Q, TKI, survival benefit, lung adenocarcinoma, polymorphism

## Abstract

The prevalence and impact of epidermal growth factor receptor (EGFR) Q787Q polymorphism on the treatment of lung adenocarcinoma remains unclear. We retrospectively analyzed patients with stage IV lung adenocarcinoma to evaluate the prevalence of the EGFR Q787Q polymorphism and its influence on effects of tyrosine kinase inhibitor (TKI) treatment. A total of 333 patients were included in this study. The prevalence of the EGFR Q787Q polymorphism was 38%, 42%, and 35% in the total patients, EGFR mutation negative, and EGFR mutation positive groups, respectively. The prevalence of EGFR Q787Q polymorphism was significantly higher in EGFR wild-type patients than in the general non-cancerous population from Taiwan Biobank and 1000 Genome Project databases, respectively. EGFR Q787Q polymorphism had significant protective effects on the overall survival of EGFR-mutant lung adenocarcinoma treated with EGFR TKIs (aHR =0.61, p=0.03). Our study demonstrated that EGFR Q787Q polymorphism is a germline variant in the general population. It is a protective predictor of overall survival in patients with stage IV EGFR-mutated lung adenocarcinoma treated with TKIs.

## Introduction

A new era of non-small cell lung cancer (NSCLC) commenced with the appearance of epidermal growth factor receptor (EGFR) tyrosine kinase inhibitors (TKIs) (1). Detection of activating EGFR mutations in stage IV NSCLC is currently the standard of care. The proportion of tumors harboring activating EGFR mutations that respond to TKIs varies across different ethnic groups. For example, Caucasians show a rate of 10% to 15% EGFR mutation positive in NSCLC. In Asians, the prevalence is 30% to 50% (2). The most common EGFR mutation in NSCLC is exon 19 deletion (E19d) and exon 21 L858R point mutation (L858R), which accounts for approximately 85% of all EGFR mutations. TKIs show better treatment effects for these two mutations (3). However, other EGFR mutations may still be sensitive to TKI treatment. As the availability of next-generation sequencing increases, rare EGFR mutations have attracted the attention of oncologists (4).

EGFR Q787Q polymorphism has been studied and classified as a rare form of EGFR mutation (4–6), and was first regarded as a candidate EGFR mutation sensitive to TKIs. However, EGFR Q787Q polymorphism is not a somatic mutation in cancer but is rather a germline variant that exists in a section of the non-cancerous population. It was coded as rs1050171 (G to A) and has a different prevalence across ethnic groups.

The nucleotide site of the Q787Q polymorphism is in the EGFR gene and has been hypothesized to be a prognostic factor for TKI treatment (7). EGFR Q787Q polymorphism is a poor prognostic factor for OS in colorectal cancer patients treated with an anti-EGFR antibody (8). However, evidence of the prognostic value of EGFR Q787Q polymorphism for TKI treatment in NSCLC remains limited and controversial (7, 9, 10).

In this study, we compared the prevalence of EGFR Q787Q polymorphism between the general and lung cancer populations. Finally, we retrospectively analyzed the prevalence of EGFR Q787Q polymorphism in stage IV lung adenocarcinoma and its association with the outcome of lung adeno-carcinoma patients treated with TKIs.

## Materials And Methods

### Study Population

The prevalence of EGFR Q787Q polymorphism in the general population was extracted from the 1000 Genome Project Database (https://www.internationalgenome.org/) and the Taiwan Biobank (https://www.biobank.org.tw/). In lung cancer patients, this retrospective study included consecutive patients with stage IV lung adenocarcinoma who were diagnosed in the Mackay Memorial Hospital in Taiwan from January 1^st^, 2016 to May 31^st^, 2019. Patients with double primary malignancies were excluded from the study. During this period, all tumors from patients with stage IV lung adenocarcinoma were tested for EGFR mutations, with EGFR Q787Q polymorphism being reported routinely. The study protocol was approved, and all methods were performed in accordance with the relevant guidelines and regulations of the Institutional Review Board of Mackay Memorial Hospital (IRB no. 21MMHIS218e), Taipei, Taiwan.

### Measurements

We retrospectively reviewed the medical records of the patients. Baseline characteristics including age, sex, smoking history, and Eastern Cooperative Oncology Group (ECOG) performance status before treatment were collected. EGFR genotyping reports were investigated, and EGFR mutation status, including EGFR Q787Q polymorphism status, was recorded. A positive EGFR mutation refers to mutations that are sensitive to TKIs. The EGFR Q787Q genotypes heterozygous G/A and homozygous A/A were identified as EGFR Q787Q polymorphism. The EGFR genotyping was performed in the tumor tissue from biopsy. The sanger sequencing method was used to detect the EGFR mutation and EGFR Q787Q polymorphism. The overall survival (OS) was calculated as the interval between the date of pathological diagnosis and death or the last follow-up date. The time to treatment failure (TTF) for TKIs was also analyzed for EGFR-mutated patients who received TKI treatment and was considered as the time from the first date of TKI treatment to the date of discontinuation or death. The last follow-up date of this study for OS and TTF was December 31^st^, 2020.

### Statistical Analysis

Odds ratios were estimated using logistic regression. Comparisons of basic characteristics were performed using the Wilcoxon signed-rank test for continuous variables and Fisher’s exact test for categorical variables. The survival curves for both OS and TTF were estimated using the Kaplan–Meier (KM) method, and the multiple Cox proportional hazards regression model was performed to estimate adjusted hazard ratios (aHRs) with covariates EGFR Q787Q polymorphism, age, sex, presence of E19d, history of smoking, ECOG performance status, presence of brain metastasis, and TKI generation, respectively, for patients with positive EGFR mutation and treated with TKI as first-line therapy. The OS for patients with negative EGFR mutations who were treated with first-line chemotherapy was estimated by the KM method and the covariates for multiple Cox proportional hazards regression model were EGFR Q787Q polymorphism, age, sex, history of smoking, ECOG performance status, and presence of brain metastasis. All tests were two-sided and p values < 0.05 were considered statistical significance. All analyses were performed using the R version 4.0.2.

## Results

### Prevalence of the EGFR Q787Q Polymorphism

During the study period, 336 consecutive patients were diagnosed with stage IV lung adenocarcinoma in our hospital. Three patients with two cancer types were excluded from the study. Of the remaining 333 cases, 201 had EGFR mutation-positive (EGFRm-positive) cancers. Among the EGFR mutation-negative (EGFRm-negative) patients, 13 had ALK and ROS1 rearrangement or met 14 skipping. There were 168 patients with a positive EGFR mutation status who received TKI treatment as first-line therapy. Among EGFRm-negative patients, 76 received first-line chemotherapy (**Supplementary Table 1**).

**Figure 1:**
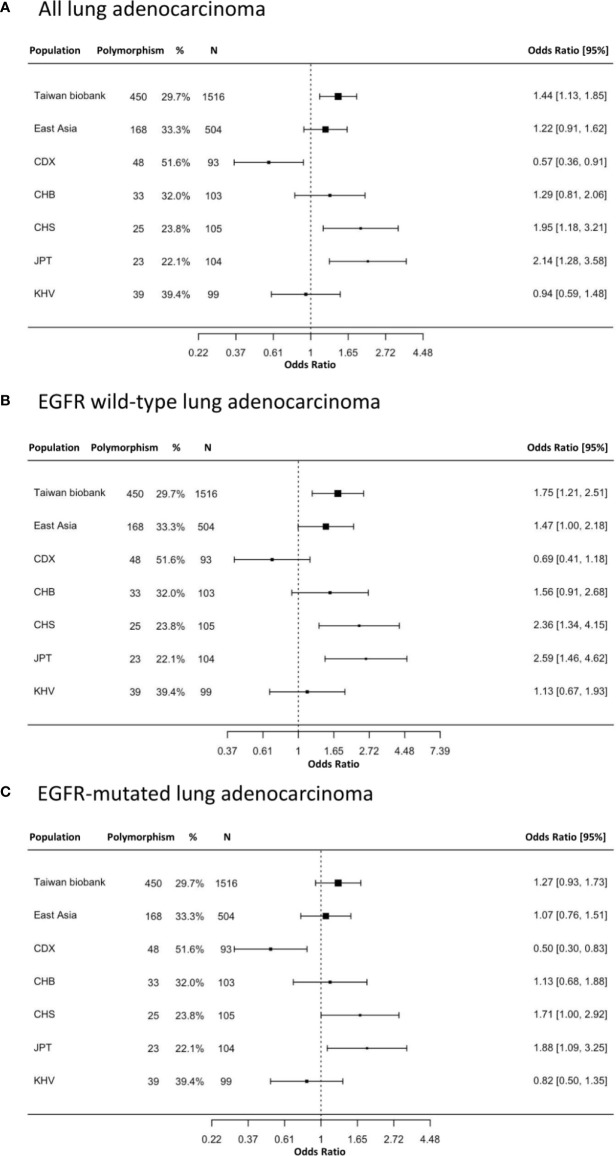
The prevalence and odds ratios of EGFR Q787Q polymorphism in lung adenocarcinoma patients and general populations. **(A)** all lung adenocarcinoma **(B)** EGFR wild type lung adenocarcinoma **(C)** EGFR-mutated lung adenocarcinoma. 95% CI: 95% confidence interval; Taiwan Biobank (n=1,516); CDX, Chinese Dai in Xishuangbanna, China (n=93); CHB, Han Chinese in Beijing, China (n=103); CHS, Southern Han Chinese, China (n=105); JPT, Japanese in Tokyo, Japan (n=104); KHV, Kinh in Ho Chi Minh City, Vietnam (n=99). The x-axis was displayed as log transform scale and corresponding numbers of odds ratio.

The prevalence of the EGFR Q787Q polymorphism which is the prevalence of heterozygous G/A and homozygous A/A genotypes was different across ethnicities (**Supplementary Table 2**). Europeans showed a high prevalence (84.5%), while East Asians (33.3%) and Taiwanese people (29.7%) had a lower prevalence. The prevalence was 38%, 42%, and 35% in the total patients, EGFR wild-type, and EGFR-mutated groups, respectively. The prevalence of the EGFR Q787Q polymorphism in the normal population was 29.7%, according to data from the Taiwan Biobank. The odds ratios for lung adenocarcinoma with the EGFR Q787Q polymorphismwere 1.44 (95% CI=1.13-1.85; p=0.004), 1.75 (95% CI=1.21-2.51; p=0.003), and 1.27 (95% CI=0.93-1.73; p=0.14) for all patients, EGFR wild-type and EGFR-mutated lung adenocarcinoma, respectively (**Figure 1**).

### EGFRm-Positive Patients With TKI Treatment

The clinicopathological characteristics of EGFRm-positive patients treated with TKIs and with or without the EGFR Q787Q polymorphism are shown in **Table 1**. The results showed no significant differences between EGFR Q787Q polymorphism statuses.

**Table 1:** Clinicopathological characteristics of TKI-treated EGFRm-positive patients.

	With EGFR Q787Q polymorphism (%) (n = 111)	Without EGFR Q787Q polymorphism (%) (n = 57)	p-value
Age (sd)	66.2 (11.7)	65.9 (12.1)	0.892
Gender (M)	34 (30.6%)	19 (33.3%)	0.729
Smoking (ever)	21 (18.9%)	11 (19.3%)	1
ECOG performance status (>1)	30 (27.0%)	16 (28.1%)	1
E19d	48 (43.2%)	27 (47.4%)	0.627
L858R	57 (51.4%)	27 (47.4%)	0.745
Brain metastasis	39 (35.1%)	17 (29.8%)	0.604
Liver metatstasis	16 (14.4%)	12 (21.1%)	0.282
Bone metastasis	52 (46.8%)	34 (59.6%)	0.143
TKI			0.535
First generation	75 (67.6%)	38 (66.7%)	
Second generation	36 (32.4%)	18 (31.6%)	
Third generation	0 (0%)	1 (1.8%)	

**Figure 2:**
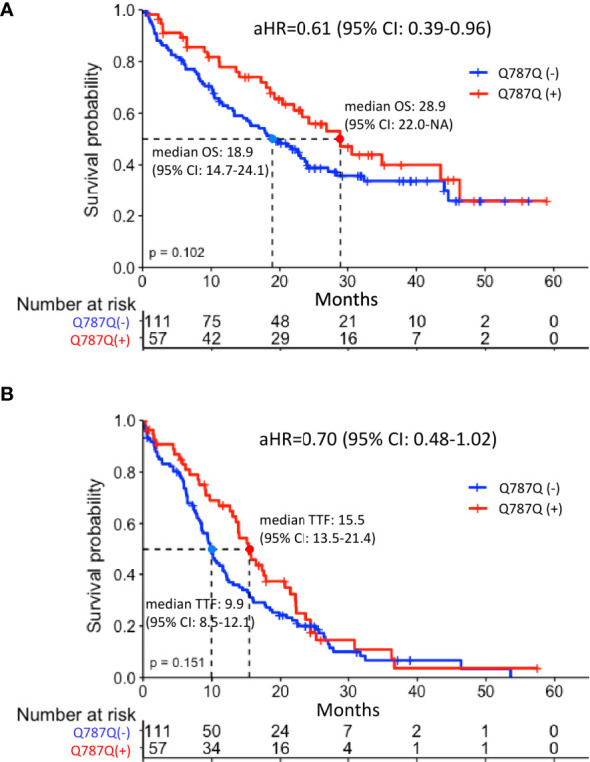
Survival analysis of EGFRm-positive patients with or without EGFR Q787Q polymorphism treated with TKI **(A)** overall survival **(B)** Time to treatment failure. Median overall survival of the groups with and without EGFR Q787Q polymorphism were 28.8 months (95% CI: 22.0-NA) and 18.8 months (95% CI: 14.6-24), respectively. Median time to treatment failure of the groups with and without EGFR Q787Q polymorphism groups were 15.5 months (95% CI: 13.5-21.4) and 9.9 months (95% CI: 8.5-12.1), respectively. Q787Q (+) and Q787Q (–) denoted patients with EGFR Q787Q polymorphism and without EGFR Q787Q polymorphism, respectively.

Patients with EGFR Q787Q polymorphism had marginally significantly longer OS (median: 28.8 months vs. 18.8 months, p=0.102) (**Figure 2A**). The KM method may have introduced a confounding effect as it did not consider the effects of other risk factors. In order to make up for this possible confounding effect, multiple Cox proportional hazards regression analyses were performed to adjust for confounding effects. Results showed that the aHR of EGFR Q787Q polymorphism was 0.61 (95% CI=0.39-0.96, p=0.03) and it indicated that EGFR Q787Q polymorphism was a statistically independent prognostic factor (**Table 2**).

**Table 2:** Adjusted hazard ratios (aHRs) for EGFR Q787Q polymorphism and other covariates in EGFR-mutated patients treated with TKIs.

	aHR	95% CI	p-value
**Overall survival**			
Age	1.03	1.01-1.05	0.009
Gender (M)	1.18	0.72-1.94	0.51
Smoking	1.53	0.85-2.73	0.16
E19d	0.74	0.48-1.15	0.18
EGFR Q787Q polymorphism	0.61	0.39-0.96	0.03
ECOG performance status (>1)	3.94	2.54-6.11	<0.001
Brain metastasis	1.05	0.65-1.71	0.84
TKI (2^nd^ vs. 1^st^ generation)	0.49	0.30-0.80	0.004
**Time to treatment failure**			
Age	1.02	1.00-1.04	0.06
Gender (M)	1.32	0.87-2.02	0.20
Smoking	1.03	0.63-1.69	0.90
E19d	0.93	0.64-1.35	0.69
EGFR Q787Q polymorphism	0.70	0.48-1.02	0.07
ECOG performance status (>1)	2.69	1.78-4.07	<0.001
Brain metastasis	1046	0.99-2.14	0.06
TKI (2^nd^ vs. 1^st^ generation)	0.55	0.36-0.83	0.005

Regarding TTF, patients with EGFR Q787Q polymorphism did not show the significant difference with patients without EGFR Q787Q polymorphism (median: 15.4 months vs. 9.9 months, p=0.15) (**Figure 2B**) and showed a marginally significant prognosis (aHR = 0.7; 95% CI=0.48-1.02; p=0.07) (**Table 2**).

Furthermore, EGFRm-negative patients with the EGFR Q787Q polymorphism did not have the OS benefit for chemotherapy as 1st line treatment (median: 6.70 months vs. 8.58 months, p=0.971) (**Supplementary Figure 1**) (aHR = 1.23; 95% CI=0.68-2.25; p=0.496) (**Supplementary Table 4**).

## Discussion

Our study demonstrated that the EGFR Q787Q polymorphism is a prognostic factor for EGFR-mutated lung adenocarcinoma treated with TKIs. In the overall survival, the survival benefit of the EGFR Q787Q polymorphism was marginally significant (median: 28.8 months vs. 18.8 months, p=0.1). However, after considering the confounding factors in the multivariable Cox model, the benefit was significant (aHR = 0.61, p=0.03). To our knowledge, only a few studies have investigated the impact of the EGFR Q787Q polymorphism on TKI effects in NSCLC. In contrast with our findings, Leichsenring et al. concluded that the EGFR Q787Q polymorphism was not a prognostic factor for lung adenocarcinoma regardless of EGFR mutation status (7). However, ethnic differences must be considered when interpreting Q787Q studies. Leichsenring et al. found a 70.6% prevalence of the EGFR Q787Q polymorphism in EGFR-mutated tumors of German patients, which is much higher than that in our cohort, where the prevalence was comparable with that of the general population (**Supplementary Table 2**). Although they analysed 102 EGFR-mutated lung cancer patients, only 18 non- EGFR Q787Q polymorphism cases were included in the survival analysis of EGFR mutants because of the high prevalence of the EGFR Q787Q polymorphism. This may reduce the significance of the results of this study. Furthermore, they included 23% of patients without metastasis in the survival analysis, which would further influence the results. Another early study from Japan showed a tendency toward worse outcomes in the EGFR Q787Q polymorphism among patients treated with gefitinib, contrary to our findings (9). However, this study was conducted early, when the prognostic value of EGFR mutations is still under investigation. The authors examined only exon 20 from the genes of patients treated with gefitinib. The most common mutations in exons 19 and 21 were not considered, causing a large prognostic bias. To the best of our knowledge, our study had the largest cohort among studies on the prognostic value of the EGFR Q787Q polymorphism in TKI-treated lung adenocarcinoma.

Here, we also showed a higher prevalence of the EGFR Q787Q polymorphism in stage IV lung adenocarcinoma than that in the general population (OR= 1.44, p=0.004), especially in wild-type EGFR cases. This result was compatible with Zhang et al.’s study (11) of 122 lung cancer patients and 147 controls, reporting a 2.67 (95% CI: 1.17–6.08) odds ratio of the EGFR Q787Q polymorphism in lung cancer. EGFR Q787Q polymorphism should be regarded as a germline variant according to the biobank databases. The prevalence of this polymorphism differs between ethnic groups, with the lowest being East Asian (33.3%) and the highest being European (84.5%). This polymorphism is not rare, even in East Asians. The rs1050171 polymorphism has another missense variant that has the nucleotide C instead of G or A, causing a change in the transcription of the amino acid from Q(Gln) to H(His). However, the EGFR Q787Q polymorphism did not cause changes in the amino acids. Therefore, it is not reasonable to treat the EGFR Q787Q polymorphism as a driver, missense, or rare mutation. A study by Quan et al. examined EGFR mutations in NSCLC from 354 Chinese patients (6). Sanger sequencing was used to identify mutations and found a 3.11% frequency of the EGFR Q787Q somatic mutations, a result that should be interpreted with reservations. First, despite the claimed detection of a somatic mutation, the authors did not mention how they distinguish somatic mutations from germline mutations. Second, if the authors did not perform further work to identify the somatic mutation, the percentage of Q787Q should be much higher than what they reported because of the high prevalence of background germline variant In a study on exon 20 mutations in EGFR, the authors detected a high percentage of the EGFR Q787Q polymorphism (rs1050171 (c.2361 A>G)) in peripheral blood samples from NSCLC patients after chemotherapy. The authors mistakenly treated the EGFR Q787Q polymorphism as a point mutation caused by chemotherapy and concluded that chemotherapy may induce EGFR-TKI resistant mutations (12). In fact, the high percentage of the EGFR Q787Q polymorphism originates from the background germline variant

In EGFR wild-type stage IV lung adenocarcinoma, we did not find a prognostic effect for the EGFR Q787Q polymorphism. In squamous cell lung cancer, patients with the EGFR Q787Q polymorphism have been reported to have poorer outcomes for stage I and II disease (10). The EGFR Q787Q polymorphism was also identified as a poor prognostic factor in advanced squamous cell carcinoma and metastatic colon cancer treated with an anti-EGFR antibody (8). The underlying mechanism by which the EGFR Q787Q polymorphism affects the disease is still not fully understood. Tan et al. conducted a promising study to illustrate how the EGFR Q787Q polymorphism influences TKI sensitivity in head and neck squamous cell cancers. They found that TKI sensitivity increased up to 70-fold in squamous cell cancers with the EGFR Q787Q polymorphism. They demonstrated that the EGFR Q787Q polymorphism caused the instability of EGFR-AS1 long non-coding RNA, which led to the high expression of EGFR isoform D and resulted in a better response to TKIs (13). This mechanism may be applicable to the explanation of our findings on lung adenocarcinoma. The mRNA and EGFR proteins of tumor cells should be further studied. However, this is beyond the scope of this study. Further investigation is needed to illustrate the complex relationship between the EGFR Q787Q polymorphism and lung cancer pathophysiology.

This study had several limitations. First, it was retrospective and there may still be bias even though we considered important confounding factors. Second, we included only patients with stage IV lung adenocarcinoma. The odds ratio of the EGFR Q787Q polymorphism could refer to advanced disease, but not all lung adenocarcinomas. Third, because the prevalence of the EGFR Q787Q polymorphism from Taiwan Biobank data was summary data instead of the individual data, the age and smoking history were not matched. It would introduce the potential bias and overestimate the odds ratio. Fourth, the tumor tissue was sequenced by the routine sanger sequence and did not compare it with the normal tissue or blood DNA. The EGFR Q787Q polymorphism cannot be distinguished from the possible somatic mutation. The estimation of the prevalence of EGFR Q787Q polymorphism in patients with lung cancer could be overestimated. Fifth, during the study period, the EGFR mutation was detected by Sanger sequencing, which has a much lower sensitivity than NGS (14). Although there should be no issues in detecting the germline EGFR Q787Q polymorphism, detecting other mutations such as *de novo* T790M may be influenced by bias. In fact, *de novo* T790M mutations were rare in our cohort and detected in only three out of 333 patients. Finally, most of our patients did not undergo re-biopsy after disease progression. We could not demonstrate the rate of T790M mutation after disease progression, which is now an effective treatment. Since the Q787Q codon of EGFR was located near the mutation site of T790M, the impact of the EGFR Q787Q polymophism on T790M is important for the discussion of the prognostic value of the EGFR Q787Q polymorphism.

## Conclusions

Our study demonstrated that EGFR Q787Q polymorphism is a germline variant with a high prevalence in the general population and should not be treated as a rare mutation. EGFR Q787Q polymorphism was associated with better survival in patients with stage IV lung adenocarcinoma treated with TKIs.

## Data Availability Statement

The original contributions presented in the study are included in the article/**Supplementary Material**. Further inquiries can be directed to the corresponding author.

## Ethics Statement

The studies involving human participants were reviewed and approved by The Institutional Review Board of Mackay Memorial Hospital, Taipei, Taiwan. Written informed consent for participation was not required for this study in accordance with the national legislation and the institutional requirements.

## Author Contributions

Conceptualization, W-JW, S-HY, H-PC, and H-YC. Data curation, W-JW, S-HY, H-PC, C-TY, and Y-TC. Formal analysis, W-JW. Investigation, S-HY and H-YC. Methodology, W-CC and H-YC. Project administration, W-JW and H-YC. Supervision, JS and H-YC. Validation, W-CC, JS, and H-YC. Writing – original draft, W-JW. Writing – review & editing, S-HY. All authors contributed to the article and approved the submitted version.

## Funding

Academia Sinica Major Diseases Grand Challenge Program (MD-GCP) AS-GC-110-MD06. Next-Generation Pathway of Taiwan Cancer Precision Medicine Program (AS-KPQ-107-TCPMP). Key and Novel Therapeutics Development Program for Major Diseases (AS-KPQ-111-KNT).

## Conflict of Interest

The authors declare that the research was conducted in the absence of any commercial or financial relationships that could be construed as a potential conflict of interest.

## Publisher’s Note

All claims expressed in this article are solely those of the authors and do not necessarily represent those of their affiliated organizations, or those of the publisher, the editors and the reviewers. Any product that may be evaluated in this article, or claim that may be made by its manufacturer, is not guaranteed or endorsed by the publisher.

## Supplementary Material

Supplementary Figure 1 KM curve for patients without EGFR mutation and chemotherapy treatment. Median overall survival: Q787Q (+): 6.70 months (95%CI: 4.75-NA); Q787Q (-): 8.58 months (95% CI: 6.14-13.2). Log rank test p=0.971. Q787Q (+) and Q787Q (-) denoted patients with EGFR Q787Q polymorphism and without EGFR Q787Q polymorphism, respectively.
