# Supplementary figures and images for: EGFR Q787Q Polymorphism Is a Germline Variant and a Prognostic Factor for Lung Cancer Treated With TKIs
Source: Front Oncol. 2022 Mar 21;12:816801. doi: 10.3389/fonc.2022.816801 (PMC8978303; doi:10.3389/fonc.2022.816801)

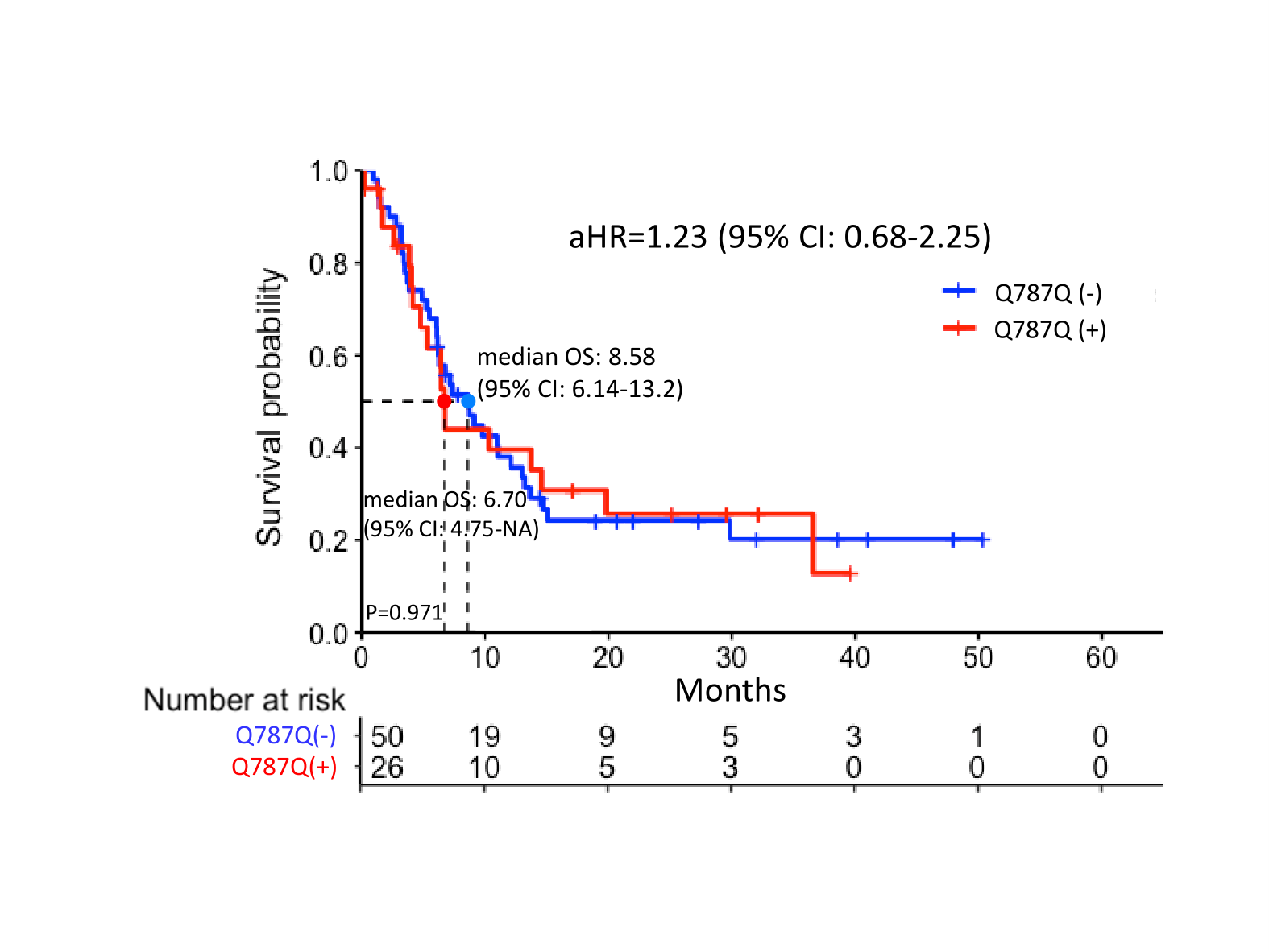

Supplement: Supplementary Figure 1 — KM curve for patients without EGFR mutation and chemotherapy treatment. Median overall survival: Q787Q (+): 6.70 months (95%CI: 4.75-NA); Q787Q (-): 8.58 months (95% CI: 6.14-13.2). Log rank test p=0.971. Q787Q (+) and Q787Q (-) denoted patients with EGFR Q787Q polymorphism and without EGFR Q787Q polymorphism, respectively. [file Image_1.tiff]
