# Supplementary material for: EGFR Q787Q Polymorphism Is a Germline Variant and a Prognostic Factor for Lung Cancer Treated With TKIs
Source: Front Oncol. 2022 Mar 21;12:816801. doi: 10.3389/fonc.2022.816801 (PMC8978303; doi:10.3389/fonc.2022.816801)
Supplement: Supplementary file 2 [file Table_1.docx]

**Supplementary Table 1.** Detailed first-line treatment.

| **Mutation type** | **Number (%)** |
| --- | --- |
| Mutation type | |
| EGFRm-positive | 201 (60.4) |
| EGFRm-negative | 132 (39.6) |
| ALK or ROS1 | 13 (3.9） |
| First line treatment type | |
| EGFRm-positive + TKI treatment | 168 (50.5) |
| EGFRm-negative + chemotherapy  EGFRm-negative + immunotherapy  ALK treatment  ROS1 treatment  EGFRm-positive + chemotherapy  EGFRm-negative + TKI  No treatment or lost follow up | 76 (22.8)  5 (1.5)  6 (1.8)  1 (0.3)  11 (3.3)  5 (1.5)  61 (18.3) |
