# Supplementary material for: EGFR Q787Q Polymorphism Is a Germline Variant and a Prognostic Factor for Lung Cancer Treated With TKIs
Source: Front Oncol. 2022 Mar 21;12:816801. doi: 10.3389/fonc.2022.816801 (PMC8978303; doi:10.3389/fonc.2022.816801)
Supplement: Supplementary file 3 [file Table_2.docx]

**Supplementary Table 2.** Prevalence of EGFR Q787Q polymorphism in the general population of different races.

|  | **With EGFR Q787Q polymorphism (%)** | **Without EGFR Q787Q polymorphism (%)** |
| --- | --- | --- |
| African (n=661) | 66.3% | 33.7% |
| American (n=347) | 79.0% | 21.0% |
| East Asian (n=504) | 33.3% | 66.7% |
| European (n=503) | 84.5% | 15.5% |
| South Asian (n=489) | 67.1% | 32.9% |

Data was from Ensembl data base.
