# Supplementary material for: EGFR Q787Q Polymorphism Is a Germline Variant and a Prognostic Factor for Lung Cancer Treated With TKIs
Source: Front Oncol. 2022 Mar 21;12:816801. doi: 10.3389/fonc.2022.816801 (PMC8978303; doi:10.3389/fonc.2022.816801)
Supplement: Supplementary file 4 [file Table_3.docx]

**Supplementary Table 3.** Patient characteristics.

|  | **Whole Population**  **(n=333)** | **EGFRm-positive**  **(n=201)** | **EGFRm-negative**  **(n=132)** |
| --- | --- | --- | --- |
| Age (sd) | 66.3 (12.0) | 66.2(11.9) | 66.5 (12.3) |
| Gender (M) | 163 (48.9%) | 53 (31.5%) | 87 (65.9%) |
| Smoking | 111 (33.3%) | 41 (20.4%) | 70 (53.0%) |
| ECOG performance status (>1) | 113 (33.9%) | 64 (31.8%) | 49 (37.1%) |
| EGFR Q787Q polymorphism | 126 (37.8%) | 70 (34.8%) | 56 (42.4%) |
| Brain metastasis | 106 (31.8%) | 67 (33.3%) | 39 (29.5%) |
| Liver metastasis | 50 (15.0%) | 33 (16.4%) | 17 (12.9%) |
| Bone metastasis | 161 (34.8%) | 103 (51.2%) | 58 (43.9%) |
