# Supplementary material for: EGFR Q787Q Polymorphism Is a Germline Variant and a Prognostic Factor for Lung Cancer Treated With TKIs
Source: Front Oncol. 2022 Mar 21;12:816801. doi: 10.3389/fonc.2022.816801 (PMC8978303; doi:10.3389/fonc.2022.816801)
Supplement: Supplementary file 5 [file Table_4.docx]

**Supplementary Table 4.** COX for OS in patient with EGFR mutation negative and chemotherapy treatment.

|  | **HR** | **95% CI** | **p-value** |
| --- | --- | --- | --- |
| Age | 1.02 | 0.99-1.04 | 0.203 |
| Gender (M) | 1.18 | 0.61-2.26 | 0.630 |
| smoking | 1.55 | 0.86-2.78 | 0.142 |
| EGFR Q787Q polymorphism | 1.23 | 0.68-2.25 | 0.496 |
| ECOG performance status (>1) | 1.35 | 0.72-2.56 | 0.351 |
| Brain metastasis | 1.00 | 0.56-1.82 | 0.988 |
